# Statin-use and perceptions of high cholesterol as predictors of healthy lifestyle behaviours in Nigerians

**DOI:** 10.1371/journal.pgph.0000190

**Published:** 2022-07-20

**Authors:** Joyce F. Coker, Kate M. Hill, Akaninyene A. Otu, Allan House

**Affiliations:** 1 Cambridge Public Health, School of Clinical Medicine, University of Cambridge, Cambridge, United Kingdom; 2 Leeds Institute of Health Sciences, University of Leeds, Leeds, United Kingdom; 3 Department of Internal Medicine, University of Calabar, Calabar, Cross Rivers State, Nigeria; Babcock University, NIGERIA

## Abstract

It is unclear how statin-use influences the adoption of healthy lifestyle choices. It is important to understand the nature of this relationship as this could facilitate targeted public health interventions which could help promote a healthy lifestyle, curb the rise of non-communicable diseases, and facilitate overall health. This study aimed to explore whether statin-use influenced the adoption of healthy lifestyle choices by changing the way urban and semi-urban Nigerians thought about their high cholesterol and their future risk of cardiovascular disease. Structured questionnaires were used to compare the lifestyle behaviours, perceptions of high cholesterol and future risk of cardiovascular disease of statin users and non-statin users recruited in urban and a semi-urban Nigeria. In-depth, face-to-face interviews were used to further explore the relationship between statin-use and the adoption of healthy lifestyle choices, and explore the influence of personal and social factors on this relationship. The odds of adopting a low-fat diet increased as perceived statin-effectiveness increased (OR = 2.33, p<0.05), demonstrating a synergistic relationship between statin-use and the adoption of healthy of lifestyle choices. In addition to this synergistic association, at interview, two other relationships were found between statin use and the adoption of healthy lifestyle choices: an antagonistic relationship fuelled by a strong perception of statin effectiveness and a perceived inability to make healthy lifestyle changes, which favoured statin-use, and an antagonistic relationship fuelled by congruous cause-control beliefs and concerns about medication-use which favoured the adoption of healthy lifestyle choices. The odds of adopting a low-fat diet was 5 times greater in urban dwellers than in semi-urban dwellers (p<0.01). Statin-use influenced the adoption of healthy lifestyle choices in three different ways, which require exploration at clinical consultation. Gender, social obligations, and physical environment also influenced statin-use and the adoption of healthy lifestyle choices.

## Introduction

Developing countries, particularly those in sub-Saharan Africa face a double burden of non-communicable and communicable diseases. The latter is still the major cause of adult mortality in the region. However, non-communicable diseases pose a substantial burden and appear to be on the rise, further straining already over-burdened and fragile health care systems [1–3]. In Nigeria, non-communicable diseases account for 29% of adult deaths, 11% of which are due to cardiovascular disease [4]. Almost half of these deaths occurred in the prime of life [5].

The rise of cardiovascular disease and other non-communicable disease in sub-Saharan Africa has been attributed to an aging population and urbanisation [6–10]. A key strategy for the primary prevention of cardiovascular disease is the lowering of lipid levels [2, 11]. This can be achieved by making lifestyle modifications such as eating a low-fat diet, regular physical activity and smoking cessation. Where needed, lipid-lowering medications are prescribed for use alongside lifestyle modifications [11–13]. Due to their documented effectiveness in lipid modification, statins have become the lipid-lowering medication of choice and one of the most prescribed medications in the world [14, 15]. However, there remains some doubt about their benefit for the primary prevention of cardiovascular disease where even relatively low rates of side effects may not be justified by their effect on preventing uncommon events [16–18].

There is sparse and conflicting data available on the relationship between statin-use and the adoption of healthy lifestyle choices [19]. Some researchers claim that statin-use provides a false sense of security, enabling people to neglect lifestyle modifications and continue to make poor lifestyle choices [20–22]. Others have either found no difference or found that statin users consumed less dietary fat than non-statin users [19, 23]. As statin-use increases, it is important to assess whether there is an interaction between statin-use and the adoption of healthy lifestyle choices for a variety of reasons. Firstly, whilst research confirms that statins produce a moderate reduction in cardiovascular disease risk, their effect is enhanced when combined with lifestyle modifications [24–26]. Secondly, these lifestyle modifications are beneficial not only for the control of lipid levels, but also for the control of other cardiovascular disease risk factors, non-communicable diseases, and promote overall health and wellbeing [5, 8, 27]. Thirdly, understanding factors that influence modifiable health-related behaviours such as the adoption of healthy lifestyle choices may facilitate the development and implementation of targeted public health interventions [2, 28–30]. This could potentially help curb the rise of cardiovascular disease risk factors in sub-Saharan Africa.

We have not identified any studies that investigate the relation between statin-use and the adoption of healthy lifestyle choices in sub-Saharan Africa where the burden of cardiovascular disease risk factors is on the increase. Consequently, this study aimed to explore (i) whether statin-use influenced the adoption of healthy dietary and exercise choices and (ii) whether it did so, by changing the way urban and semi-urban Nigerians perceived their high cholesterol and their future risk of cardiovascular disease. To fulfil these research aims, the following 4 research questions were explored:

1. Does statin-use influence the adoption of healthy dietary and exercise choices?
2. Does statin-use influence the way urban and semi-urban Nigerians think about their high cholesterol and their future risk of cardiovascular disease?
3. Do perceptions of high cholesterol and perceptions of future risk of cardiovascular disease influence the adoption of healthy dietary and exercise choices?
4. How do personal and social factors influence statin-use, perceptions of high cholesterol and future risk of cardiovascular disease, and the adoption of healthy dietary and exercise choices?

The Common Sense Model of self-regulation, and the Health Belief Model were used as frameworks to understand how illness perceptions and health beliefs influence health-related behaviours [28, 31–33]. The Common Sense Model posits that when faced with a health threat, individuals produce a cognitive representation of the health threat which informs their choice of coping strategies [33]. The Health Belief Model was originally developed to explain or predict preventive health behaviours in healthy individuals [32]. Both models acknowledge the modifying role of socio-cultural factors on illness perceptions.

## Materials and methods

### Study design

#### Mixed methods approach

This study employed an explanatory sequential mixed methods approach. This approach involved the collection and analysis of data in 2 distinct phases. Phase 1 involved the collection and analysis of quantitative data. The findings of phase 1 were used to inform the choice of participants purposefully sampled to take part in phase 2—a qualitative study. Both studies were analysed separately and integrated in the discussion section [34].

#### Quantitative research methods

A structured questionnaire was used to obtain information about participants’ demographics, cardiovascular risk factors, dietary and exercise behaviours, perceptions of their high cholesterol, and future risk of cardiovascular diseases.

#### Qualitative research methods

In-depth interviews carried out in-person were used to explore the narratives voiced by statin users and non-statin users about their dietary and exercise behaviours, perceptions of high cholesterol, and perceived future risk of cardiovascular diseases. These interviews were also used to explore participants’ accounts of how personal and social factors influenced their aforementioned behaviours and perceptions.

### Study setting

This study was conducted across two sites in Nigeria. The first site was the Nigeria National Petroleum Corporation (NNPC) medical services located in the cosmopolitan Maitama district in Abuja, Nigeria. NNPC is a national oil and gas company and provides free healthcare for its staff and their immediate family. Abuja is the capital of Nigeria and has been exposed to a lot of Western influences. This site will be referred to as the urban research site. The second site was the University of Calabar Teaching Hospital (UCTH) located in Calabar, the capital of Cross-River state in the oil-rich Niger delta region. Tourism has become an avenue of wealth creation for Cross-River state [35]. Consequently, Cross River state is rapidly undergoing urbanisation [36]. This site will be referred to as the semi-urban research site. Healthcare at this site was not provided free of charge.

### Ethical approval and informed consent

Ethical approval was obtained from the Nigerian Institute for Medical Research (IRB/13/216), from the local research ethics committees of both research sites (GGM/11/12 & UCTH/LR/DM/16), and from the Leeds Institute of Health Sciences and Leeds Institute of Genetics, Health and Therapeutics and Leeds Institute of Molecular Medicine joint ethics committee (HSLTLM/12/063 R).

Prior to completion of the questionnaire, the researcher verbally went through and provided all participants with an information sheet which detailed the voluntary nature and purpose of the study, details of participation, and freedom to and process of withdrawal from the study. Written consent was not requested for questionnaire completion. Rather, the return of self-completed questionnaires, or the act of following the researcher into a vacant consultation room, and verbally completing researcher-administered questionnaires were deemed as implied consent. Before the commencement of all interviews, the researcher verbally went through the information sheet again with participants and written consent was sought before the recorder was switched on and interviews begun.

### Study sample

Adults aged 18-years and older who had a recorded diagnosis of hyperlipidaemia and attended a hospital appointment at either research site between August to October 2013 were invited to take part in the study. People were excluded from the study if they: (i) did not speak English; (ii) had experienced a diagnosed cardiovascular event—in accordance with our focus on the primary prevention of cardiovascular disease and; (iii) had a diagnosis of familial hypercholesterolemia or type 1 diabetes due to the significant role of genetics in these conditions.

The respondents’ age for statin and non-statin users were 52.7±10.5 and 53.2±11.9 years, respectively. Participants were predominantly females (57%), had hypertension (60%), and 24% had hypertension and diabetes (Table 1). Over half (55%) of participants were recruited from the semi-urban site. Urban dwellers were significantly better educated than semi urban dwellers (tertiary education = 79.4% urban dwellers vs 38.3% semi urban dwellers, Fisher’s exact = 41.14, p<0.001), and were more likely to have lived outside of Nigeria in the 10-years prior to this study than semi-urban dwellers (OR = 7.5, X^2^ = 12.72, p<0.001).

**Table 1:** Characteristics of quantitative study sample.

Variable	Statin users n = 78 (52.7%)	Non-statin users n = 70 (47.3%)	Overall n = 148 (100%)
**Mean age (years)** (SD)	52.68 (10.45)	53.16 (11.88)	52.91 (11.11)
20–29	0 (0)	2 (2.9)	2 (1.4)
30–39	9 (11.5)	4 (5.7)	13 (8.8)
40–49	21 (26.9)	20 (28.6)	41 (27.7)
50–59	27 (34.6)	25 (35.7)	52 (35.1)
60–69	19 (24.4)	13 (18.6)	32 (21.6)
70–79	1 (1.3)	4 (5.7)	5 (3.4)
80–89	1 (1.3)	2 (1.4)	3 (2.0)
**Gender**			
Male	35 (44.9)	29 (41.4)	64 (43.2)
Female	43 (55.1)	41 (58.6)	84 (56.8)
**Site of recruitment**			
Semi-urban	39 (50.0)	43 (61.4)	82 (55.4)
Urban	39 (50.0)	27 (38.6)	66 (44.6)
**Marital status**			
Single	6 (7.7)	6 (8.6)	12 (8.1)*
Married	62 (79.5)	53 (75.7)	115 (77.7)*
Separated/ divorced	7 (9.0)	1 (1.4)	8 (5.4)*
Widowed	3 (3.8)	9 (12.9)	12 (8.1)*
Unknown	0 (0)	1 (1.4)	1 (0.7)
**Education**			
Primary or less	21 (26.9)	26 (37.1)	47 (31.8)
Secondary level	11 (14.1)	5 (7.1)	16 (10.8)
Tertiary level	44 (56.4)	37 (52.9)	81 (54.7)
Unknown	2 (2.6)	2 (2.9)	4 (2.7)
**Lived outside Nigeria in past 10 years**			
Yes	11 (14.1)	7 (10.0)	18 (12.2)
No	67 (85.9)	62 (88.6)	129 (87.2)
Unknown	0 (0)	1 (1.4)	1 (0.7)
**Ethnicity**			
Cross Rivers	35 (44.9)	42 (60.0)	77 (52.0)*
Hausa and Fulani	8 (10.3)	0 (0)	8 (5.4)*
Igbo	12 (15.4)	11 (15.7)	23 (15.5)*
Ijaw	6 (7.7)	2 (2.9)	8 (5.4)*
Yoruba	10 (12.8)	5 (7.1)	15 (10.1)*
Other	6 (7.7)	9 (12.9)	15 (10.1)*
Unknown	1 (1.3)	1 (1.4)	2 (1.4)
**Hypertension**			
Yes	49 (62.8)	39 (55.7)	88 (59.5)
No	18 (23.1)	18 (25.7)	36 (24.3)
Unknown	11 (14.1)	13 (18.6)	24 (16.2)
**Diabetes**			
Yes	31 (39.7)	23 (32.9)	54 (36.5)
No	35 (44.9)	32 (45.7)	67 (45.3)
Unknown	12 (15.4)	15 (21.4)	27 (18.2)
**Hypertension and diabetes**			
Yes	19 (24.4)	17 (24.3)	36 (24.3)
No	47 (60.3)	38 (54.3)	85 (57.4)
Unknown	12 (15.4)	15 (21.4)	27 (18.2)

* p≤0.05

### Recruitment strategy

Eligible participants were approached by the researcher in the waiting room. The researcher verbally went through the information sheet and obtained verbal consent prior to the administration of the questionnaires. In the semi-urban site, all questionnaires were researcher-administered. In the urban research site, participants were given the option to self-administer their questionnaires and return them to the researcher or have their questionnaires administered by the researcher.

A purposive sample of participants who completed the questionnaires were invited to take part in in-depth interviews and written consent was sought. The intention was to recruit a minimum of 4 participants from each of the 4 categories below:

1. Statin users who had adopted a low fat diet and/or healthy exercise behaviours
2. Statin users who had not adopted a low fat diet and/or healthy exercise behaviours
3. Non-statins users who had adopted a low fat diet and/or healthy exercise behaviours
4. Non-statin users who had not adopted a low fat diet and/or healthy exercise behaviours.

All interviews took place in a vacant consultation room where only the interviewee and the researcher were present. All interviews were audio-recorded and subsequently transcribed verbatim.

### Quantitative data collection

#### Statin status (explanatory variable)

Participants who were taking a statin at the time of the study were classified as statin users. Non-statin users, included participants who had not been prescribed a statin, and participants who had previously been prescribed a statin but were not taking a statin at the time of the study.

#### Demographics and cardiovascular disease risk factors (explanatory variables)

A structured questionnaire was used to elicit demographic information from participants. A medical diagnosis of diabetes, hypertension and statin-use at the time of the study were confirmed by participants prior to the administration of the questionnaire.

#### Perception of high cholesterol and future risk of cardiovascular disease (explanatory variables)

The Revised Illness Perception Questionnaire (IPQ-R) was used to assess participants’ perceptions of their high cholesterol [37]. The identity or symptom sub-scale was not included in this study because high cholesterol is asymptomatic [31]. Two items “spiritual causes” and “fate/destiny” were added to the cause subscale of the IPQ-R because religion plays a major role in the way Nigerians perceive the world around them and make sense of illness, death and suffering [38].

Champion’s Health Belief Model Scale was used to assess the participants’ beliefs about their future risk of cardiovascular disease [39]. The health motivation sub-scale was not included in this study as it is not one of the 4 core components of the health belief model [32, 40].

#### Adoption of a low fat diet and healthy exercise choices (primary outcome variables)

Prochaska and DiClemente’s model of change was used to assess adoption of a low-fat diet and adoption of healthy exercise choices [41]. Participants classified by this model as belonging to either the pre-contemplation, contemplation or decision stages of change for either health behaviour were regarded as non-adopters of the relevant health behaviour (low fat diet, healthy exercise choices). Participants in the action or maintenance stages of change were regarded as adopters of the relevant health behaviour [41].

#### Dietary and exercise behaviours (secondary outcome variables)

Questions from the European Prospective Investigation of Cancer and Nutrition food frequency questionnaire were used to elicit information about the dietary fat consumption [42, 43]. The options of cooking oil in the study questionnaire were altered to reflect the Nigerian diet.

Questions from the UK Department of Health’s General Practice Physical Activity Questionnaire were used to assess participants’ level of physical activity and classify them as either: inactive, moderately inactive, moderately active or active [44].

To ensure that the questionnaire was understandable and culturally acceptable, it was piloted on 10 Nigerians aged between 27-65years.

### Quantitative data analysis

Data from the questionnaires was entered into and analysed using the Statistical Package for Social Sciences version 20. Frequencies were calculated for categorical variables and mean scores were calculated for each of the sub-scales in the Revised Illness Perception Questionnaire and Champion’s Health Belief Model Scale [37, 39, 45]. In accordance with the instructions for the Revised Illness Perception Questionnaire, the total score and not mean score of each cause subscale was calculated [37]. Chi-square tests or Fisher’s exact tests were used to assess between group differences in categorical variables. Independent t-tests or Mann-Whitney tests were used to assess between group differences in continuous variables.

Logistic regression models were used to identify variables that were associated with the adoption of a low-fat diet and the adoption of healthy exercise choices. The minimum sample size required for this study was calculated on the premise that 15 participants were required for each predictor variable included in the regression model [46, 47]. Therefore, the minimum sample size required to perform a reliable logistic regression with 5 predictor variables was 75 participants. An increase in the number of participants recruited was matched with an increase in the number of variables included in the regression model. For all statistical tests, p<0.05 was taken as the level of statistical significance.

### Qualitative data collection

The researcher conducted face-to-face, semi-structured interviews with participants in a vacant consultation room. Existing literature was used to develop an interview guide which helped elicit information from interviewees about the influence of statin-use on: (i) their adoption of a low-fat diet and/or healthy exercise behaviours; (ii) their perception of high cholesterol; and (iii) their perception of the future risk of cardiovascular disease. The interview guide was piloted on a small sample of Nigerians aged between 27-65years. All interviews were audio recoded, lasted between 30–90 minutes and were anonymised and transcribed verbatim by the researcher (JC) who is familiar with the use and interpretation of Pidgin English and Nigerian slang words.

### Qualitative data analysis

All interview transcripts were coded using the NVivo 10 software package. Transcripts were re-read to ensure transcription was verbatim and to familiarise researchers with the interview data. Braun and Clark’s 6 phases of thematic analysis were used to guide the analysis of interview data and ensure a rigorous thematic analysis was conducted [48].

The initial coding process was inductive and focused on identifying themes that were emerging from the data. The research questions were not at the forefront of the coding process. However, an awareness of the various components of the Common Sense Model guided the choice of theme names. To ensure that the coding process was consistent, the emerging codes were used to generate a codebook. Codes were agreed upon, and collated into themes, which in turn were categorised into clusters by JC, KH and AH.

## Quantitative results

### Dietary patterns

Most participants (76%) reported that they ate fried foods at home on a weekly basis, while only 35% reported doing so away from their homes. As shown in Table 2, 69% of participants reported that they had adopted a low-fat diet. Over half of these participants (59%) had been doing so for a least a year prior to the study. Statin and non-statin users did not significantly differ in their reported adoption of a low-fat diet or any other reported dietary behaviours.

**Table 2:** Dietary patterns of quantitative study sample.

Variable	Statin users n = 78 (52.7%)	Non-statin users n = 70 (47.3%)	Overall n = 148(100%)
**Weekly fried food consumption at home**			
Yes	59 (75.6)	54 (77.1)	113 (76.4)
No	18 (23.1)	16 (22.9)	34 (23.0)
Unknown	1 (1.3)	0 (0)	1(0.7)
**Weekly fried food consumption outside the home**			
Yes	30 (38.5)	22 (31.4)	52 (35.1)
No	48 (61.5)	47 (67.1)	95 (64.2)
Unknown	0 (0)	1 (1.4)	1(0.7)
**Think current diet is low fat?**			
Yes	51 (65.4)	38 (54.3)	89 (60.1)
No	20 (28.2)	23 (32.9)	43 (29.1)
Unknown	7 (9.0)	9 (12.9)	16 (10.8)
**Ever decreased fat in diet?**			
Yes	62 (79.5)	53 (75.7)	115 (77.7)
No	16 (20.5)	17 (24.3)	33 (22.3)
**Currently decreasing fat in diet?**			
Yes (Adopters)	57 (73.1)	45 (64.3)	102 (68.9)
No (Non-adopters)	20 (25.6)	25 (35.7)	45 (30.4)
Unknown	1 (1.3)	0 (0)	1 (0.7)
**Adopters only**			
**Duration of decreasing fat in diet**			
<30 days	4 (7.0)	3 (6.7)	7 (6.9)
1–6 months	12 (21.1)	11 (24.4)	23 (22.5)
7–12 months	7 (12.3)	2 (4.4)	9 (8.8)
>1 year	32 (56.1)	28 (62.2)	60 (58.8)
Unknown	2 (3.5)	1 (2.2)	3(2.9)
**Non-Adopters only**			
**Considered decreasing dietary fat in the past month?**			
Yes	0 (0)	3 (12.0)	3 (6.7)
No	19 (95.0)	17 (68.0)	36 (80.0)
Unknown	1 (5.0)	5 (20.0)	6 (13.3)

Urban dwellers were significantly more likely to report that they ate fried foods on a weekly basis than semi-urban dwellers (65% vs 91%, X^2^ = 15.61, p<0.001). The odds of reporting the consumption of fried foods outside the home was 16 times higher in urban dwellers than in semi-urban dwellers (11% vs 65%, Fisher’s exact = 51.41, p<0.001). Nevertheless, the odds of reportedly adopting a low-fat diet was 6 times higher in urban dwellers than in semi-urban dwellers (55% vs 86%, X^2^ = 18.30, p<0.001). They were also significantly more likely to reportedly think they were eating a low-fat diet than semi-urban dwellers (44% vs 80%, X^2^ = 21.90, p<0.001).

### Exercise patterns

As shown in Table 3, Majority of participants (80%) were classified as physically inactive/moderately inactive, reported that they had never increased the frequency/intensity of their exercise (78%), and were classified as non-adopters of healthy exercise behaviours (85%). Statin users were significantly less inactive/moderately inactive than non-statin users (86% vs 74%, X^2^ = 3.852, p = 0.05).

**Table 3:** Physical activity and exercise patterns of quantitative study sample.

Variable	Statin users n = 78 (52.7%)	Non-statin users n = 70 (47.3%)	Overall n = 148(100%)
**Physical activity level**			
Inactive/moderately inactive	67 (85.9)	52 (74.3)	119 (80.4)*
Moderately active/active	10 (12.8)	18 (25.7)	28 (18.9)
Unknown	1 (1.3)	0 (0)	1 (0.7)
**Ever increased frequency/intensity of exercise?**			
Yes	18 (23.1)	14 (20.0)	32 (21.6)
No	60 (76.9)	56 (80.0)	116 (78.4)
**Currently doing more exercise?**			
Yes (adopters)	13 (16.7)	11 (15.7)	24 (16.2)
No (non-adopters)	65 (83.3)	59 (84.3)	124 (83.8)
**Adopters only**			
**Duration of increasing frequency/intensity of exercise**			
<30 days	6 (46.2)	1 (9.1)	7(29.2)
1–6 months	3 (23.1)	4 (36.4)	7 (29.2)
7–12 months	0 (0)	1 (9.1)	1 (4.2)
>1 year	4 (30.8)	5 (45.5)	9 (37.5)
**Non-adopters only**			
**Considered increasing frequency/intensity of exercise in the past month?**			
Yes	13 (20.0)	16 (27.1)	29 (23.4)
No	52 (80.0)	40 (71.2)	94 (75.8)
Unknown	0 (0)	1 (1.7)	1 (0.8)

* p≤0.05

Urban and semi-urban dwellers did not significantly differ in their levels of physical activity or reported adoption of healthy exercise behaviours. However, significantly more urban dwellers reported that they had at some point in time increased their frequency/intensity of exercise (OR = 2.5, 30% vs 15%, X^2^ = 5.30, p = 0.027). Urban dwelling non-adopters were also 15 times more likely to report that they had considered adopting healthy exercise behaviours in the previous month than semi-urban dwelling non-adopters (48% vs 6%, X^2^ = 30.00, p<0.001).

### Perception of high cholesterol

As shown in Table 4, participants reportedly believed their high cholesterol could be controlled by their own behaviours ($\bar{x}$ = 3.9, SD = 0.62) and by taking a statin ($\bar{x}$ = 3.7, SD = 0.64). However, perceptions of the behavioural control were stronger than perceptions of statin control. Correspondingly, the most important cause of high cholesterol reported by participants was lifestyle causes, followed by biomedical causes. The low score obtained on the timeline acute/chronic subscale, indicates that participants perceived their high cholesterol as an acute condition.

**Table 4:** Perceptions of high cholesterol.

Variables	Statin	Non-statin	Overall
	users	users	
	n = 75 (SD)	n = 64 (SD)	n = 139 (SD)
Mean perceived timeline acute/chronic	2.12 (0.67)	2.31 (0.70)	2.21 (0.66)
Mean perceived timeline cyclical	3.16 (0.84)	3.18 (0.85)	3.17 (0.84)
Mean perceived consequences	2.94 (0.68)	2.94 (0.65)	2.94 (0.66)
Mean perceived personal control	3.87 (0.68)	3.84 (0.58)	3.85 (0.62)
Mean perceived statin control	3.78 (0.66)	3.57 (0.59)	3.68 (0.64) **
Mean perceived emotional response	2.95 (0.95)	3.03 (0.93)	2.99 (0.94)
Mean perceived illness coherence	3.24 (1.13)	3.19 (1.13)	3.22 (1.12)
Total perceived biomedical cause£	13.99 (2.90)	14.06 (3.91)	14.02 (3.39)
Total perceived spiritual cause€	7.08 (3.37)	6.55 (2.98)	6.83 (3.20)
Total perceived lifestyle cause$	23.51 (4.35)	23.97 (4.86)	23.72 (4.58)

**p≤0.01, £ this sub-scale has 5 items and scores range between 5–25, € this subscale has 3 items and scores range between 3–15, $ this subscale has 8 items and scores range between 8–40.

Statin users and non-statin users reported stronger perceptions of personal control than statin control of high cholesterol. This indicates that both groups reportedly believed that their behaviours could control their high cholesterol better than statin-use. However, statin users reported significantly stronger statin control perceptions than non-statin users ($\bar{x}$ = 3.8 vs $\bar{x}$ = 3.6, U = 1721.500, p = 0.003).

Urban dwellers reportedly thought that they had a poor understanding of high cholesterol compared to semi-urban dwellers ($\bar{x}$ = 3.6 vs $\bar{x}$ = 2.9 U = 1497.000, p<0.001). They also reported significantly stronger perceptions that high cholesterol is an acute condition ($\bar{x}$ = 2.0 vs $\bar{x}$ = 2.4 U = 1587.500, p<0.001), but they perceived it to be less predictable i.e. less cyclical than semi-urban dwellers ($\bar{x}$ = 2.9 vs $\bar{x}$ = 3.4, U = 1626.500, p = 0.002).

### Perception of future risk of cardiovascular disease

As shown in Table 5, participants reportedly perceived healthy lifestyle choices ($\bar{x}$ = 4.00, SD = 0.65) to be more beneficial than statin-use for the prevention of cardiovascular disease ($\bar{x}$ = 3.8, SD = 0.70). However, they thought there were more barriers to making healthy lifestyle choices than to taking a statin. The low score obtained on the perceived susceptibility sub-scale indicates that participants did not perceive themselves to be at risk of cardiovascular disease.

**Table 5:** Perceptions of future risk of cardiovascular disease.

Variable	Statin	Non-statin	Overall
	users	users	
	n = 71 (SD)	n = 62 (SD)	n = 133 (SD)
Perceived susceptibility	2.44 (0.84)	2.25 (0.80)	2.35 (0.83)
Perceived severity	3.10 (0.68)	2.86 (0.75)	2.99 (0.72)*
Perceived benefits of statins	3.88 (0.67)	3.72 (0.73)	3.80 (0.70)
Perceived benefits of healthy lifestyle choices	4.00 (0.65)	4.01 (0.66)	4.00 (0.65)
Perceived barriers to statins	2.31 (0.72)	2.56 (0.79)	2.43 (0.76)*
Perceived barriers to healthy lifestyle choices	2.76 (0.76)	2.69 (0.74)	2.73 (0.75)

* p≤0.05

Statin users reportedly perceived significantly fewer barriers to statin-use for cardiovascular disease prevention ($\bar{x}$ = 2.3 vs $\bar{x}$ = 2.6, U = 1715.000, p = 0.027), and perceived cardiovascular disease to be significantly more severe than non-statin users ($\bar{x}$ = 3.1 vs $\bar{x}$ = 2.9, U = 1745.500, p = 0.040).

Urban dwellers reportedly perceived cardiovascular disease to be significantly more severe than semi-urban dwellers ($\bar{x}$ = 3.2 vs $\bar{x}$ = 2.8, U = 1707.500, p = 0.027). They also thought they were fewer barriers to ($\bar{x}$ = 2.0 vs $\bar{x}$ = 2.8, U = 1815.000, p = 0.840) and significantly more benefits of the adoption of healthy lifestyle for cardiovascular disease prevention than semi-urban dwellers ($\bar{x}$ = 4.1 vs $\bar{x}$ = 3.9, U = 1635.500, p = 0.007).

### Factors associated with the adoption of a low-fat diet

Of the 148 participants recruited, 86% completed all sections of the questionnaire that were entered into a logistic regression model where the dependent variable was adoption of a low-fat diet and independent variables were: statin-status, gender, research site, physical activity level, perceived statin control of high cholesterol, perceived barriers to statin-use for cardiovascular disease prevention, and perceived severity of cardiovascular disease.

The logistic regression model was statistically significant (X^2^ = 25.822, p = 0.001) and correctly classified 73% of cases. The model explained between 18.3% (Cox and Snell R^2^) and 26.0% (Nagelkerke R^2^) of variance in the adoption of a low-fat diet, this highlights the importance of exploring the role of other factors using qualitative research methods. The Hosmer and Lemeshow test of fit indicated that the model was a good fit to the data (X^2^ = 6.00, p = 0.647).

As shown in Table 6, only research site and perceived statin control of high cholesterol made statistically significant contributions to the model. The odds of adopting a low-fat diet was 5 times greater in participants recruited from the urban research site and increased as reported perceived statin control of high cholesterol increased.

**Table 6:** Logistic regression model for the adoption of a low-fat diet.

Variables	B	S.E	Wald	P value	OR	95%CI
Statin status (statin-use)	-0.20	0.46	0.20	0.66	0.82	0.34–1.96
Gender (Male)	0.45	0.48	0.87	0.35	1.56	0.61–4.00
Research site(semi-urban)	-1.51	0.52	8.90	0.003**	0.21^$^	0.08–0.59^$^
Physical activity level (inactive/moderately inactive)	-0.20	0.60	0.11	0.74	0.82	0.25–2.66
Statin control of high cholesterol	0.85	0.36	5.60	0.018**	2.33	1.16–4.69
Barriers to statins-use to prevent CVD	-0.33	0.30	1.24	0.27	0.72	0.40–1.29
Perceived severity of CVD	0.41	0.38	1.19	0.28	0.28	0.72–3.16

*p ≤0.05

**p≤ 0.01

$ Inverted version of these values i.e. 1 divided by the value is presented in the text to make interpretation easier. n = 128.

The model assessing factors associated with the adoption of healthy exercise behaviours was not statistically significant (X^2^ = 9.674, p = 0.378) and will not be presented in this article.

## Qualitative results

Eight participants were interviewed; 4 statin users (3 classified as non-adopters in quantitative study) and 4 non-statin users (1 non-adopter). The characteristics of interviewees are presented in Table 7.

**Table 7:** Characteristics of interviewees in qualitative study.

Participants	Recruitment site	Healthy lifestyle adoption classification from quantitative study
Statin users		
Male 1	Semi-urban site	Non-adopter
Male 2	Semi-urban site	Diet and exercise adopter
Female 1	Semi-urban site	Non-adopter
Female 5	Semi-urban site	Non-adopter
Non-statin users		
Female 2	Semi-urban site	Diet only adopter
Female 3	Semi-urban site	Non-adopter
Female 4	Semi-urban site	Diet only adopter
Female 6	Urban site	Diet and exercise adopter

**Fig 1:**
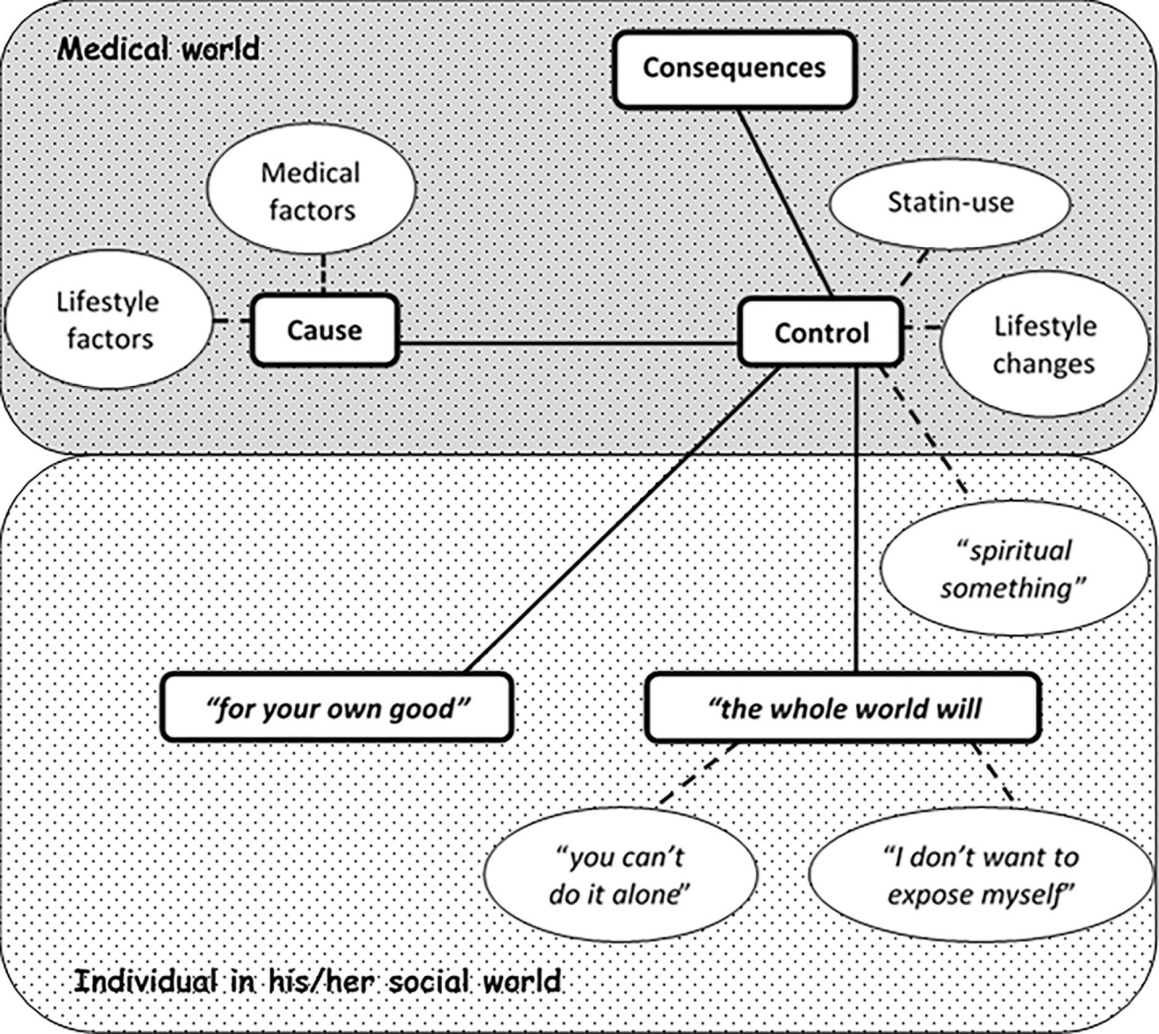
Thematic map.

Five main themes emerged from the analysis of participant interviews, namely: (i) consequences, (ii) cause, (iii) control, (iv) *“for your own good”*, and (v) “*the whole world will talk”*. As shown in Fig 1, these themes clustered into 2 areas of discussion, namely: discussions about the medical world of high cholesterol, and discussions about the participant as an individual in his/her social world. Themes regarding the medical world of high cholesterol were named according the aspect of the high cholesterol under discussion. Themes regarding the individual in his/her social world were named using a phrase participants’ themselves used. Each theme and subsequent subthemes are described below. Relevant interview extracts are presented in Table 8.

**Table 8:** Interview extracts from the qualitative study.

Theme	Quotation
Theme 1: Consequences	
	*“the consequences*, *if I am not mindful there are a lot of consequences if am not mindful… If I drink excessively*, *eh*, *if I don’t do exercise*, *if I don’t check my (stammers) levels of cholest (stammers)*, *whatever*, *it can*, *you know*, *I must have to be very careful*, *I know my diet*.*”* **Male 2** Interviewer: *What do you think can happen if you don’t*? *I could (stammers) stroked*. *God forbid*. *Eh if am not mindful*, *heart attack will come in”* **Male 2**
Theme 2: Cause	
Medical Lifestyle	*“I believe it was it could be a hereditary factor at that level*, *at that level cos at 30 I’m not supposed to have high cholesterol and be hypertensive*. *I know that my dad died of hypertension but at the age of sixty something ehh*, *but me it has shown clear that hereditary factor was into play and more so am not the first son*.*”* **Male 2** *“What caused it is maybe too much of taking cholesterol*, *taking carbohydrates in excess*, *then maybe late eating"* **Female 4**
Theme 3: Control	
No control Statin-use Lifestyle control *“spiritual something”*	*“I have never tried to control it…I like what I do*, *I like how I have been and nobody has the right to complain to me cause it’s my body”* **Male 1** *“You know the most important thing is to come to the hospital for regular check*. *Because when it is discovered that you have that [high cholesterol]*, *you could be placed on drug because you know to me simtab [simvastatin] is very good*. *It’s the best and can lower the cholesterol in diabetic patients and regular check-up too”* **Female 5** *“Exercise can’t…*. *Medication is good*, *dieting is good*. *Exercise is not all that good for them [cardiovascular disease risk factors] …Cos your health does not need something that will really stress them o that is what I think o*.*”* **Female 2** *“I will pray normal*, *but this prayer point is not that maybe its spiritual something*. *This is what you are taking in [eating]*, *it’s not something*, *it depends on how you can control your diet in eating aspect*. *So it’s not about prayer matter*, *it’s not about spiritual something*. *So just to know what to take in [eat]”* **Female 4**
Theme 4: “For your own good”	
	*“they [*doctors*] will now give you some medications and its good you have to take it because it’s for your own good……I have to take my medication because it is being prescribed by a doctor to take”* **Female 1** *“You know*, *once you know you are right you don’t allow someone intimidate you When I started talking*, *he[doctor] admired and I said I’m learned*, *a degree master’s holder……*.. *So he really had to school me and he also told me some implications and why he would change some drugs*. *And I now told him no*, *why you must not change this drug*. *I argued them [stammers]*. *Because I know*.*”* **Male 2**
Theme 5: “the whole world will talk”	
*“I don’t want to expose myself”* “*you can’t do it alone”*	“So none of them [friends] know am hypertensive or I have this cholesterol…I avoid telling them, it’s embarrassing, stigmatization, they will look at you, ahh you mean [says own surname], you are. . .until I married my wife she did never knew I was hypertensive or had high cholesterol” **Male 2** *“Friends can help you doing that*. *If there are around with you*. *Like exercise if you want to do exercise do it very very well*, *you have to get someone that will be gingering [*encouraging*] you*.* *.* *. *Like me I can’t do exercise alone*, *but if I see someone that will be doing it doing it I will be saying agh agh*, *how is my own different*, *I will try my best to follow that person [laughs]”* **Female 2**

### Themes 1: Consequences

Both male interviewees identified heart attacks, strokes and death as potential consequences of high cholesterol. These perceived consequences appeared to encourage statin-use and the adoption of healthy lifestyle choices as illustrated by the quote from Male 2.

### Themes 2: Cause

Interviewees attributed their high cholesterol to 2 factors, namely: (i) medical factors and (ii) lifestyle factors.

#### Sub-theme 1: Medical factors

Two interviewees (Male 2, Female 3) attributed their high cholesterol to genetics factors and recounted their family history to the interviewer without being prompted. They both cited statin-use and the adoption of healthy lifestyle choices simultaneously as a suitable strategy to manage their high cholesterol. This demonstrates a synergistic relationship between statin-use and the adoption of healthy lifestyle choices. It should be noted that Male 2 was classified as a statin-user and adopted of both a low fat diet and healthy exercise behaviours whilst Female 3 was classified as a non-statin user and non-adopter of both a low fat diet and healthy exercise behaviours.

#### Sub-theme 2: Lifestyle factors

Lifestyle factors, predominantly diet, were the most common cause of high cholesterol discussed by interviewees. Most of these participants also cited lifestyle as the best way to control their high cholesterol, demonstrating a congruous cause-control relationship. No female interviewee cited physical inactivity as a cause of their high cholesterol. Rather, they cited the consumption of sugary foods and carbohydrates, consumption of fatty foods, eating late, and increased alcohol consumption as a result of stress as the cause of their high cholesterol.

### Themes 3: Control

The control beliefs and descriptions of the changes interviewees discussed making or not making in an attempt to control their high cholesterol fall in 4 subthemes, namely: (i) no control; (ii) statin-use; (iii) lifestyle changes; and (iv) *“spiritual something”*

#### Sub-theme 1: No control

Only 2 interviewees mentioned that they made no effort to control their high cholesterol. Female 6 cited work stress as the cause of her increased alcohol consumption and poor dietary habits. She explained that she previously had no control over her busy schedule and thus was initially unable to make healthy lifestyle changes. Her work circumstances had however changed by the time of the study. Whilst she felt unable to make changes due to her perceived lack of agency, Male 1 refused to make changes because of his presence of agency. He explained that he had not made any lifestyle changes to control his high cholesterol because he was happy as he was and felt no-one should tell him what to do with his body.

#### Sub-theme 2: Statin-use

Interviewees expressed mixed statin control beliefs and demonstrated 3 different relationships between statin-use and the adoption of healthy lifestyle choices. Some interviewees voiced positive statin control perceptions, and also had very positive beliefs about regular hospital visits and medical checks. Most of these interviewees were female statin users who had not adopted a low-fat diet or healthy exercise behaviours. They also voiced weak lifestyle control perceptions because they perceived themselves as unable or struggling to adopt healthy lifestyle choices. These interviewees demonstrate an antagonistic relationship between medical control and lifestyle control of high cholesterol, in a manner that facilitated statin-use, but hindered the adoption of healthy lifestyle choices.

Similarly, most of the interviewees who expressed weak statin control perceptions, voiced strong lifestyle control perceptions, were non-statin users, and were classified as adopters of a low-fat diet and/or healthy exercise behaviours. These interviewees demonstrate an antagonistic relationship between medical control and lifestyle control of high cholesterol in a manner that hindered statin-use but facilitated the adoption of healthy lifestyle choices. Their negative statin control beliefs were fuelled by concerns about the side-effects of statin use, dislike of lifelong medication use, and the belief that their high cholesterol was caused by lifestyle factors, and therefore should be controlled by lifestyle factors (congruous cause-control perceptions).

Only 1 interviewee voiced positive statin control and lifestyle control perceptions simultaneously and demonstrated a synergistic relationship between statin use and the adoption of healthy lifestyle choices. He (Male 2) attributed his high cholesterol to genetic factors, believed statin-use and lifestyle changes could control his high cholesterol, and was classified as a statin user, and an adopter of both a low-fat diet and healthy exercise behaviours.

#### Sub-theme 3: Lifestyle changes

Most interviewees described attempting to make a variety of dietary changes, such as: reducing their consumption of fatty foods, fast foods, sugary foods, fizzy drinks, alcohol, salt and carbohydrates. They also discussed changing their meal times and improving their food purchasing habits. Only a few interviewees discussed attempting to adopt healthy exercise behaviours. Those who did, mentioned that they tried to walk or jog more. There were also mentions of swimming, skipping and using a treadmill.

Male interviewees believed that they could make any lifestyle changes they chose to make. Female interviewees however, described several barriers to adopting healthy lifestyle choices and recounted more barriers to make healthy exercise changes than to adopting healthy dietary choices. They cited the pleasure they derived from eating, and the time demands of work life and family obligations, as factors that hindered their adoption of healthy dietary choices. These time demands were also said to hinder their adoption of healthy exercise behaviour, as well as: the body aches and pains that accompany exercise, physical limitations as a result of other comorbidities, not feeling like exercising, the constant commitment required to keep the weight off, and the belief that exercise is in itself stressful and could have adverse health effects as illustrated by the quote from Female 3 in Table 8.

#### Sub-theme 4: “Spiritual something.”

Mutual to all interviewees was the belief that forces greater than themselves had the ability to impact their lives. They all either discussed the role of prayer, God and/or expressed fatalistic beliefs. Several interviewees described using prayer alongside medical and/or lifestyle control strategies. A few of whom stated that prayer, whilst important to them, should not usurp statin-use and/or the adoption of healthy lifestyle choices. Fatalistic beliefs however, appeared to hinder statin-use and the adoption of healthy lifestyle choices. The belief that life is unpredictable or predestined, appeared to allow interviewees to externalise control of their high cholesterol. It also diminished their perceptions of the consequences of high cholesterol potentially hindering statin-use and/or the adoption healthy lifestyle choices.

### Themes 4: “For your own good”

This theme discusses the personal factors interviewees cited as facilitators of, or barriers to their statin-use and adoption of healthy lifestyle choices. These factors appear to differ by gender. Majority of female interviewees described themselves as fat, heavy or healthy and expressed their desire to “*trim down”* or maintain their current weight and fitness levels. However, they emphasised that they did not want to become slim or too slim. These body image concerns and concerns for their health appeared to be the main personal factors which both encouraged and hindered their adoption of healthy lifestyle choices.

Male interviewees expressed the importance of: (i) their choice/decision, and (ii) their capability to adopt healthy lifestyle choices. They both emphasised that they were capable of making lifestyle and medication changes, if it was something they desired, or had chosen to do. They narrated instances of openly disagreeing with advice from medical professionals and refusing to adopt medication or lifestyle changes which they did not deem necessary or beneficial for themselves. In contrast, only a few female interviewees described themselves as drivers of their dietary behaviours and medication-use. Females in this study mainly discussed feeling compelled or obligated to take their statins because it had been prescribed by a doctor and was for their own good.

### Themes 5: “The whole world will talk”

This theme discusses the social pressures exerted by, and the support received from the members of the social world of interviewees which facilitated or hindered their statin-use and/or adoption of healthy choices. This theme consists of 2 themes, namely: (i) *“I don’t want to expose myself”*; and (ii) “*you can’t do it alone”*.

#### Sub-theme 1: “I don’t want to expose myself.”

Most participants narrated accounts of tailoring their behaviours to suit the expectations of members of their social world and maintain what they perceived to be a favourable public image. Male interviewees discussed hiding their health issues and adoption of healthy lifestyle, or simply not adopting healthy lifestyle choices all together in a bid to protect their public image. Female interviewees explained that losing weight may make people think they were sick, had HIV, or were experiencing life stress. They appeared to perceive being “*slim*” negatively. Rather, they said they wanted to “*just trim down”*, “*just to reduce*”, “*just to maintain shape*” and “*just want to feel healthy*”. Their choice of words seem to indicate that they only wanted to make small or minor changes and avoided losing too much weight. This illustrates how the perceptions of the ideal body image held by oneself and one’s social world hinders the adoption of healthy lifestyle choices.

#### Sub-theme 2: “You can’t do it alone.”

A few interviewees mentioned that they received social support from their parents in the form of advice on healthy living, and financial support to aid access to medical advice and the purchasing of medications.

Male interviewees described the practical and emotional support they received from their wives as a facilitator of their adoption of healthy lifestyle choices. Female interviewees cited the body image preferences of their husbands, and the time constraints of their marital and childcare obligations as factors that hindered their adoption of healthy lifestyle choices. They did however describe their desire to teach their children healthy lifestyle behaviours and ensure their family remained healthy as a facilitator of their adoption of healthy lifestyle choices.

Male and female interviewees also differed in their discussions of the role group membership/friendships played in their adoption of healthy lifestyle choices. Male interviewees described their friendship groups as a hindrance. Male 2 explained that he knew his friends were not a good influence on his healthy lifestyle, but they made him happy and he enjoyed their companionship. Female interviewees described their friendship groups as supportive. Their friendships appeared to offer them information about locally available healthy options and opportunities, as well as companionship in their journeys to adopt healthy lifestyle choices.

## Discussion

The primary aim of this study was to explore whether statin-use influenced the adoption of healthy dietary and exercise choices. The quantitative study found that statin-use in itself did not influence the adoption of a low-fat diet. However, statin-users were more physically inactive/moderately inactive than non-statin users.

### Statin-use and diet

Our quantitative study and three American studies found that statin-use was not independently associated with the adoption of a low-fat diet [19, 20, 49]. In contrast, a Swedish study found that statin-users had better dietary and exercise behaviours than non-statin users [23]. However, the statin users in the Swedish study had significantly higher cardiovascular risk profiles, had experienced more cardiovascular events than their non-statin using comparators, and were recruited from a pharmacy [23]. Consequently, they have may have received more lifestyle advice, may have stronger perceptions of the consequences of high cholesterol, and may represent an adherent population.

### Statin-use and physical activity

Our quantitative study found that significantly more statin-users were classified as physically inactive/moderately inactive than non-statin users. This contrasts with the findings of 2 American studies which found no difference in the physical activity levels of participants using lipid-lowering medications versus those not taking lipid-lowering medications [19, 49]. This contrast may have occurred because participants in this study, as identified in our qualitative study, attributed their high cholesterol to dietary factors therefore prioritised making dietary changes (congruous cause-control perceptions). Consequently, statin users may have felt that statin-use alongside the adoption of healthy dietary behaviours were adequate strategies to control their high cholesterol. This may have allowing them to neglect the adoption of healthy exercise behaviours.

It should be noted that participants in this study (statin users and non statin statin users) demonstrated a preference for making dietary changes than making exercise changes. Our quantitative study found that although majority of our participants had adopted a low-fat diet, and had done so for at least a year prior to this study, majority of participants were classified as physically inactive/moderately inactive, had never adopted healthy exercise behaviours, and had not considered doing so in the month prior to this study. Similarly, our qualitative study revealed that most interviewees discussed making healthy dietary changes but only a few recounting making any exercise changes. Research on the interplay between dietary behaviours and physical activity behaviours is inconsistent, inconclusive and limited [50, 51]. However, there is some evidence of an asymmetrical relationship between dietary and exercise behaviours, where exercise is used to compensate for, or offset poor dietary behaviours, although this is not always the case [51, 52]. Dietary behaviours therefore may be seen as the major, whilst exercise the minor behaviour. An asymmetrical diet and exercise relationship observed in this study may have co-existed or may have been fuelled by the attribution of high cholesterol to dietary factors resulting in the preferences for making healthy dietary changes than healthy exercise changes.

### Interactions between statin-use and the adoption of healthy lifestyle choices

This study also aimed to explore whether statin-use influenced the adoption of healthy lifestyle choices by changing the way urban and semi-urban Nigerians thought about their high cholesterol and their future risk of cardiovascular disease. Our quantitative study found that statin-use in itself did not significantly influence the adoption of a low-fat diet. However, our qualitative study found 3 different types of interactions between statin-use and adoption of healthy lifestyle choices: (i) a synergistic relationship between statin-use and the adoption of health lifestyle choices, (ii) an antagonist relationship between statin-use and the adoption of health lifestyle choices which favoured the statin-use, and (iii) an antagonist relationship between statin-use and the adoption of health lifestyle choices which favoured the adoption of healthy lifestyle choices. These findings and the conflicting data from other studies [19–23] suggest that the relationship between statin-use and the adoption of healthy lifestyle choices is complex and may vary depending on the context in which it occurs.

Statin-use in itself did not significantly influence the adoption of a low-fat diet in our quantitative study. However, the odds of adopting a low-fat diet increased as perceived statin control of high cholesterol increased. This is consistent with the synergistic relationship between statin-use and the adoption of health lifestyle choices identified in our qualitative study. In our qualitative study, this synergistic relationship occurred in a context where high cholesterol was not solely attributed to dietary factors. Research also offers 3 possible explanations for why some participants thought statin-use worked in unison with the adoption of healthy lifestyle choices to manage their high cholesterol. The first explanation may be that medical advice encouraging the adoption of healthy lifestyle choices occurred at the same time statins were prescribed [19]. Secondly, the prescription of a statin may increase risk perception and serves as a wake-up call which facilitates the adoption of healthy lifestyle choices [19, 23]. Thirdly, a dislike for medication-use or concern about long-term medication-use may encourage both statin-use and the adoption of healthy lifestyle choices in an attempt to lower cholesterol cease medication-use and return to optimal health [19].

The second relationship we found between statin-use and the adoption of healthy lifestyle choices was an antagonistic relationship which favoured the adoption of healthy lifestyle choices. This relationship occurred in the context where interviewees disliked long-term medication-use, were concerned about the side-effects of statin-use, and believed that high cholesterol was caused by and thus best controlled by lifestyle factors (congruous cause-control beliefs). This relationship was mainly voiced by interviewees who were non-statin users and is consistent with the quantitative finding that non-statins users had significantly weaker statin control beliefs than statin users, and perceived significantly more barriers to statin-use for the prevention of cardiovascular disease than non-statin users. It is well-documented that statin-use is frequently declined or discontinued by patients around the world because of concerns about side-effects [53, 54]. Existing studies which explored perceptions of statin-use have also found that a dislike of general medication-use not just statin-use specifically fuels a preference for adopting healthy lifestyle choices or other non-pharmaceutical methods such as the use of herbal remedies and supplements over statin-use [55–60].

The third relationship we found between statin-use and the adoption of healthy lifestyle choices was an antagonistic relationship which facilitated statin-use and hindered the adoption of healthy lifestyle choices. This relationship occurred in a context where interviewees perceived themselves as unable to adopt healthy lifestyle choices due to a weak sense of agency and/or competing priorities. This is consistent with our quantitative finding that despite being more perceived as beneficial for the prevention cardiovascular disease, the adoption of healthy lifestyle choices was thought to have more barriers than statin-use. Similarly, Mann et al. reported that some participants begun statin-use because of their perceived inability to adopt healthy lifestyle choices [20]. Statin-use thus appears to be viewed by some as an easier alternative to the adoption of healthy lifestyle choices rather than as a complementary strategy [20, 49, 61]. Another possible explanation for the preference for statin-use over the adoption of healthy lifestyle choices may be that improvements in lipid profile following the commencement of statin-use may foster/encourage the perception that cholesterol levels have/are being adequately managed by statin-use and there is no need to employ additional strategies such as the adoption of healthy lifestyle choices [49]. This is problematic as the effects of statins are enhanced by the adoption of healthy lifestyle choices [62], and healthy lifestyle behaviours are beneficial for the control non-communicable disease and overall health [5, 8, 27].

### Gender and social membership

This study also found that the factors which influenced the adoption of healthy lifestyle choices differed by gender. Female interviewees described how their desire to be trim but not too slim (body image concerns) infleunced their adoption of healthy lifestyle choices. Jemisenia et al. describe this as the desire to look plump i.e. not too fat, but not too slim [63]. Existing research evidence demonstrates a preference for plumper body sizes among females in Nigeria and many other developing countries [63–67]. It should be noted that being fat and flabby is not perceived as desirable. Rather, beauty, fertility, wealth and health are equated with being shapely [63, 65–67]. The preference for plumpness appears to hold even in females who have high educational achievements and are aware of the link between excess body weight and poor health outcomes [64]. This suggests and is consistent with the narrative among female interviewees in our study, that the cultural preference for plumpness may override the need to lose a significant amount of weight for health benefits and hinder the adoption of healthy lifestyle choices.

Female interviewees also worried that a significant amount of weight loss could taint ones public image by portraying them as unwell, having HIV/AIDS or experiencing difficult life circumstances and this hindered their adoption of healthy lifestyle choices. Indeed it is documented that in in Nigeria, plumpness is often associated with abundance, wealth and health while slimness denotes hunger, poor health, weakness, and HIV/AIDS [65, 66]. HIV/AIDS was sometimes referred to as the slim disease because of its association with significant weight-loss [68]. Male interviewees also demonstrated a desire to to protect or maintain a public image albeit slightly differently. They hid or completely disregarded health behaviours that may portray them as less manly to protect or maintain their public image. This is consistent with literature on hegemonic masculinity which cites a desire for good health, power and dominance as key features [69].

Other features of hegemonic masculinity which emerged from the male narrative as factors that influenced their adoption of healthy lifestyle choices were the ability to make decisions, control, protect and conquer [69, 70]. Hegemonic masculinity has been directly linked to health behaviours in men such as medication-use and dietary behaviours. Many men aspire to this form of masculinity because it is associated with power, assertiveness, success and health [69].

Family and partner support also differed by gender. Whilst family support facilitated of their adoption of healthy lifestyle choices in the male narrative—consistent with existing evidence of the positive influence wives can play on the lifestyle behaviours of men [71, 72]. Family in the female narrative, characterised busy schedules of tasks, obligations, and the body image preferences of their husbands was described as a hinderance to the adoption of healthy lifestyle choices. It is well-documented that in many instances females still play a leading role in childcare and home management, often alongside paid work. This limits their leisure time and ability to adopt healthy lifestyle choices particularly exercise [38, 73]. Nevertheless, the role females played in ensuring that their families were healthy was cited as a facilitator of the adoption of healthy dietary choices.

### Urban and semi-urban living and lifestyle

The factor which contributed the most to the multivariant model assessing the adoption of a low-fat diet, was the site from which participants were recruited. Indeed, the largest differences observed in the adoption of healthy lifestyle choices, and perception of high cholesterol and future cardiovascular disease risk occurred between urban dwellers and semi-urban dwellers.

The quantitative study findings reveal that urban dwellers consumed more fried foods, ate out more often, and were more likely than semi-urban dwellers to have lived outside of Nigeria in the 10-years prior to this study. This demonstrates that the urban dwellers in this study were more exposed to Western diets and had acquired more unhealthy lifestyle behaviours than semi-urban dwellers. This is consistent with research conducted in low-middle income countries which has found that the increased exposure to western diets (rich in salt, sugar and saturated fat) and the built environment of urban areas i.e. the relatively high availability of fast food outlets, and sedentary lifestyles facilitates the adoption unhealthy lifestyle choices and the development of cardiovascular disease and its risk factors [74, 75].

Nevertheless, urban dwellers were more likely than non-urban dwellers to have adopted a low-fat diet, and to consider adopting healthy exercise behaviours. This, alongside the findings that urban dwellers perceived their future cardiovascular disease risk to be more severe, and the adoption of healthy lifestyle choices to be more beneficial for the prevention of cardiovascular disease than the semi-urban dwellers, indicates that urban dwellers were more aware of the negative consequences of a westernised lifestyle and were actively trying to make healthy lifestyle choices. Urban dwellers may also have had more facilities and resources that facilitate the adoption of healthy lifestyle choices available to them than semi-urban dwellers [76].

### Study limitations

Illness perceptions often develop and change with time, and the nature of the relationship between illness perceptions and health behaviour is complex [77]. The former may influence the latter, but the latter may also feedback and influence the former [28, 78]. Collecting data at a single point in time using a quantitative questionnaire simplifies the complex relationship between illness perceptions and health behaviours. Furthermore reliance on the recall of interviewees influences the accuracy of the information obtained [79]. Consequently, no claims can be made about whether certain illness perceptions caused certain health behaviours and vice versa.

Although self-reported measures of lifestyle behaviours are commonly used, they are thought to overestimate adherence to health behaviours [80, 81]. Furthermore, recruiting participants from hospitals, conducting interviews, and collecting data in hospital using a researcher administered questionnaire, introduces social desirability in an adherent population. This could lead to increased discussions centred around the medical world of high cholesterol and an overestimation of adherence to healthy lifestyle choices.

We interviewed a very limited number of men and urban dwellers. This was because many of them had limited time to spend because they had other priorities such as picking up their prescriptions and returning to their daily routines. Their narrative may have enriched our data collection and enabled us to paint a more complete picture of the interaction between statin-use and the adoption of healthy lifestyle choices.

## Conclusion

This study found that the relationship between statin-use and the adoption of healthy lifestyle choices is complex and may vary depending on the context in which it occurs. It identified 3 relationships between statin-use and the adoption of healthy lifestyle choices and the contexts in which they occur. We found that in the context where high cholesterol was not solely attributed to dietary factors, some participants believed that statin-use and the adoption of healthy lifestyle choices worked in unison to manage their high cholesterol. In the context where there is concern about side-effects and a dislike for long-term medication some participants favoured the adoption of healthy lifestyle choices over statin-use. While in a context where participants felt unable or struggled to make healthy lifestyle choices, statin-use was perceived as an easier alternative. The latter poses a problem as the adoption of healthy lifestyle choices is beneficial not just for the management of high cholesterol, but also for the prevention and management of all non-communicable disease and overall health. Efforts should be made to identify people who may hold the latter set of beliefs and attempts should be made to help identify and tackle their perceived barriers to the adoption of healthy lifestyle choices and address their reliance on statin control for the management of their high cholesterol.

We also found that the adoption of healthy exercise choices among study participants was uncommon and the prevalence of physical inactivity was high. This highlights the need for public health interventions aimed at increasing physical activity for the prevention of non-communicable disease and improvement of overall health. Such interventions should take into account the influences of gender and physical environment on the adoption of healthy lifestyle choices.

Finally, this study found that urban dwellers were more likely to have adopted and thought about adopting healthy lifestyle choices than semi-urban dwellers. This demonstrates a growing awareness of the negative future impacts of Westernised lifestyle behaviours in urban areas yet highlights the need for public health interventions in semi-urban areas in order to curtail the adoption of unhealthy lifestyle choices and diminish the rise of cardiovascular disease in Nigeria.
